# HBx promotes cell proliferation by disturbing the cross-talk between miR-181a and PTEN

**DOI:** 10.1038/srep40089

**Published:** 2017-01-05

**Authors:** Yi Tian, Xinqiang Xiao, Xing Gong, Feng Peng, Yun Xu, Yongfang Jiang, Guozhong Gong

**Affiliations:** 1 Department of Infectious Diseases, the Second Xiangya Hospital, Central South University, Changsha 410011, China

## Abstract

Hepatitis B virus X protein (HBx) is involved in the initiation and progression of hepatocellular carcinoma (HCC). However, the mechanism is still needed to be elucidated. In this study, we explored the relationship between HBx and microRNA and their roles in hepato-carcinogenesis. Firstly, by global microarray-based microRNA profiling and qRT-PCR, we found miR-181a was strongly up-regulated in HepG2.2.15 cells (HBV positive) and pHBV1.3-expressing HepG2 cells, and HBx played a major role in it. Secondly, reduced PTEN protein expression in the presence of HBx was aslo mediated by miR-181a, and in the Luciferase reporter system, miR-181a inhibited the PTEN translation by binding the PTEN 3′-untranslated-region (UTR), and PTEN protein was decreased when epigenetic expression of miR-181a and rescued by knocking down miR-181a. Finally, HBx interrupted the balance between apoptosis and proliferation, which contributed to the development of hepatocellular carcinoma, was also related to the interaction of miR-181a and PTEN. Taken together, we presented here a novel cross-talk between miR-181a and PTEN which was raised by HBx, and this shined a new line in HBV-related hepato-carcinogenesis.

Hepatocellular carcinoma (HCC) is the fifth most commonly diagnosed cancer and the third leading cause of cancer-related death around the world^1^. Chronic hepatitis B virus (HBV) infection is the most prominent cause for HCC and high serum viral load of HBV is predictive of HCC development^2^,^3^. However, the mechanism by which HBV contributes to the development of HCC remains unclear. MicroRNAs (miRNAs) play important roles in many of the major biological processes including cell differentiation, proliferation, apoptosis, metabolism, development, and immunity in eukaryotic cells by regulating their target genes post-transcriptionally^4^. Thus, aberrant miRNA expression contributes to tumorigenesis and cancer progression^5^. MicroRNA-181a (MiR-181a) is a multifunction miRNA that participates in many biological processes such as apoptosis, cell proliferation and cellular invasion^6^,^7^. MiR-181a is critical in maintaining stemness of epithelial cell adhesion molecule (EpCAM) + AFP + hepatic cancer stem cells (HepCSCs)^8^,^9^. Moreover, *in vivo* expression studies show that miR-181a promotes tumor growth of SMMC-7721 cells in nude mice^10^. However, the regulatory mechanism and significance of elevated miR-181a in HBV–related HCC have not been fully understood.

Phosphatase and tensin homolog (PTEN) is one of the most frequently mutated tumor suppressors. PTEN is an upstream negative regulator of the survival phosphoinositide 3-kinase (PI3K)/AKT cascade; activation of the signal pathway of PI3K/AKT is frequently observed in multiple cancers due to loss of PTEN. The low expression of PTEN in HCC is associated with more aggressive biological behavior and poorer patient survival^11^.

In the present study, we sought to gain insight into the regulatory mechanisms of miR-181a and PTEN in HBV-related HCC. Our findings suggest that miR-181a is involved in the suppression of PTEN induced by HBx. We also show that aberrant expression of miR-181a is associated with HBV-related hepato-carcinogenesis through PTEN gene modulation, suggesting a possible novel therapeutic strategy.

## Results

### HBx is critical in HBV promoting miR-181a expression in hepatocyte

**Figure 1:**
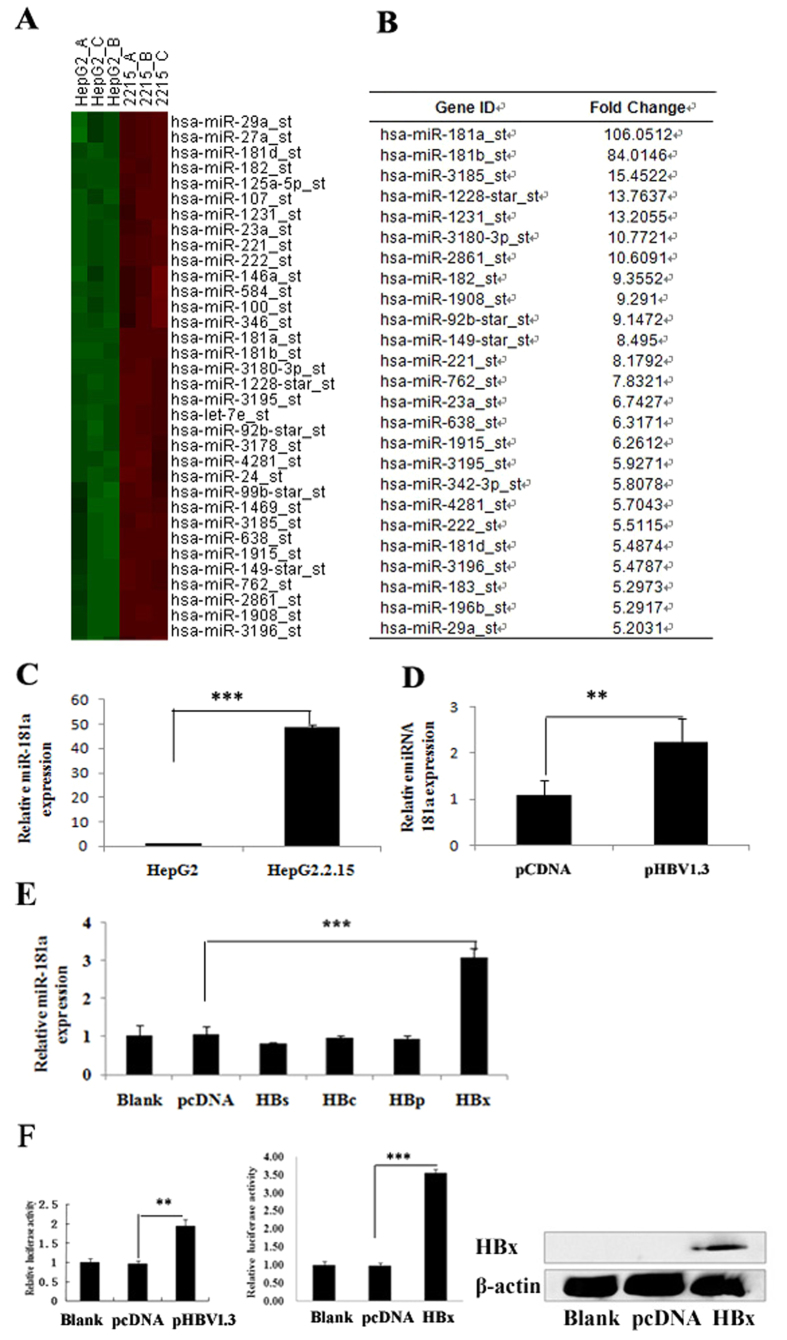
HBx is critical in HBV promoting miR-181a expression in hepatocyte. (**A**) miRNA expression heat map depicting differentially expressed miRNAs (n = 3, *p* < 0.05) in HepG2 and HepG2.2.15 cells. (**B**) A total of 62 miRNAs were up-regulated in HepG2.2.15 compared to HepG2 cells (n = 3, *p* < 0.05) (the top elevated 25 microRNAs were listed). (**C**) qRT-PCR analysis of miR-181a in HepG2 and HepG2.2.15 cells. (**D**) qRT-PCR analysis of miR-181a in HepG2 cells transfected with HBV1.3 vector and with the corresponding control (pCDNA3.0). (**E**) qRT-PCR assay for miR-181a in HBs-, HBc-, HBp-, or HBx-expressing cells and the corresponding controls. The data represent the mean ± SD. (**F**) Dual-luciferase assay for the miR-181a promoter activity of HBV or HBx-expressing cells relative to the corresponding control, versus the blank group (n = 3, *p < 0.05, ***p < 0.001).

To investigate the effect of HBV on miRNA expression, a miRNA microarray was conducted to compare the miRNA profiles between HepG2 cells and HepG2.2.15 cells which constitutively replicate HBV relative to HepG2 cells. The microarray data were analysed using hierarchical clustering of the log2 value and displayed as a heat map (Fig. 1A). Of the 615 identified miRNAs, 62 miRNAs were up-regulated and 151 miRNAs were down-regulated in the HepG2.2.15 compared to HepG2 cells. MiR-181a was most prominently expressed in HepG2.2.15 cells (Fig. 1B). We further confirmed the elevated expression of miR-181a in HepG2.2.15 cells using qRT-PCR which demonstrated that the miR-181a level in HepG2.2.15 cells was dramatically higher compared to HepG2 cells (46 fold, *p* < 0.001, Fig. 1C). The miR-181a level in HepG2 cells transiently transfected with HBV1.3 vector was also higher than that of corresponding control (Fig. 1D).

To gain insight into the biological role of HBx in miRNA expression pattern, we analyzed the expression of miR-181a in human HepG2 cells transfected with HBs, HBc, HBp and HBx vector, respectively^12^. We found that HBx was the only protein encoded by HBV that promoted miR-181a expression (Fig. 1E). To further investigate the regulatory mechanisms of HBx on miR-181a, the activity of miR-181a promoter was detected in the HepG2 cells transfected with HBV1.3, pHBx and pcDNA3.0 vector by using luciferase reporter assay. The results showed that the promoter activity of miR-181a was significantly higher in HepG2-HBV cells or HepG2-HBx cells compared with the control group (1.94 or 3.55 fold, respectively, *p* < 0.001) (Fig. 1F) which suggested that both HBV and HBx up-regulate the expression of miR-181a through enhancing its promoter activity.

### HBx is also pivotal in HBV suppressing PTEN protein expression

**Figure 2:**
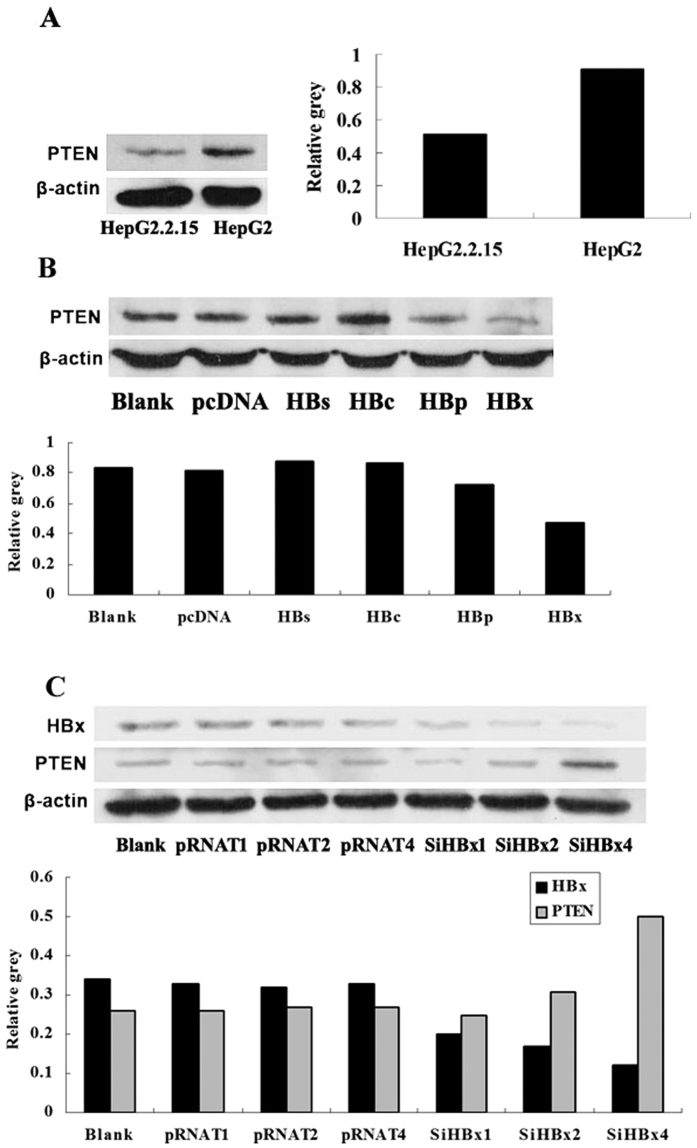
HBx is pivotal in HBV suppressing PTEN protein expression. (**A**) Western blotting analysis for PTEN in HepG2 and HepG2.2.15 cells. (**B**) Western blotting analysis for PTEN in HBs-, HBc-, HBp-, and HBx-expressing HepG2 cells and the corresponding controls, versus the blank group. (**C**) Western blotting analysis for PTEN in HepG2.2.15 cells with HBx inhibition at different doses (SiHBx vector1 μg, 2 μg, 4 μg, respectively) and the corresponding controls (pRNAT vector 1 μg, 2 μg, 4 μg, respectively), versus the blank group.

Western blot analysis revealed suppressed PTEN expression in HepG2.2.15 compared to HepG2 cells (Fig. 2A). Moreover, HBx inhibited PTEN expression more remarkably contrasted to HBs-, HBc-, HBp-transfected HepG2 cells or negative control (Fig. 2B). With HBx being suppressed by RNAi in HepG2.2.15 cells, the expression of PTEN protein promoted in a dose dependent manner (Fig. 2C). All these strongly supported that HBx is the vital protein encoded by HBV that down-regulates PTEN expression.

### MiR-181a down-regulates PTEN expression via 3′-untranslated-region (UTR) pairing

**Figure 3:**
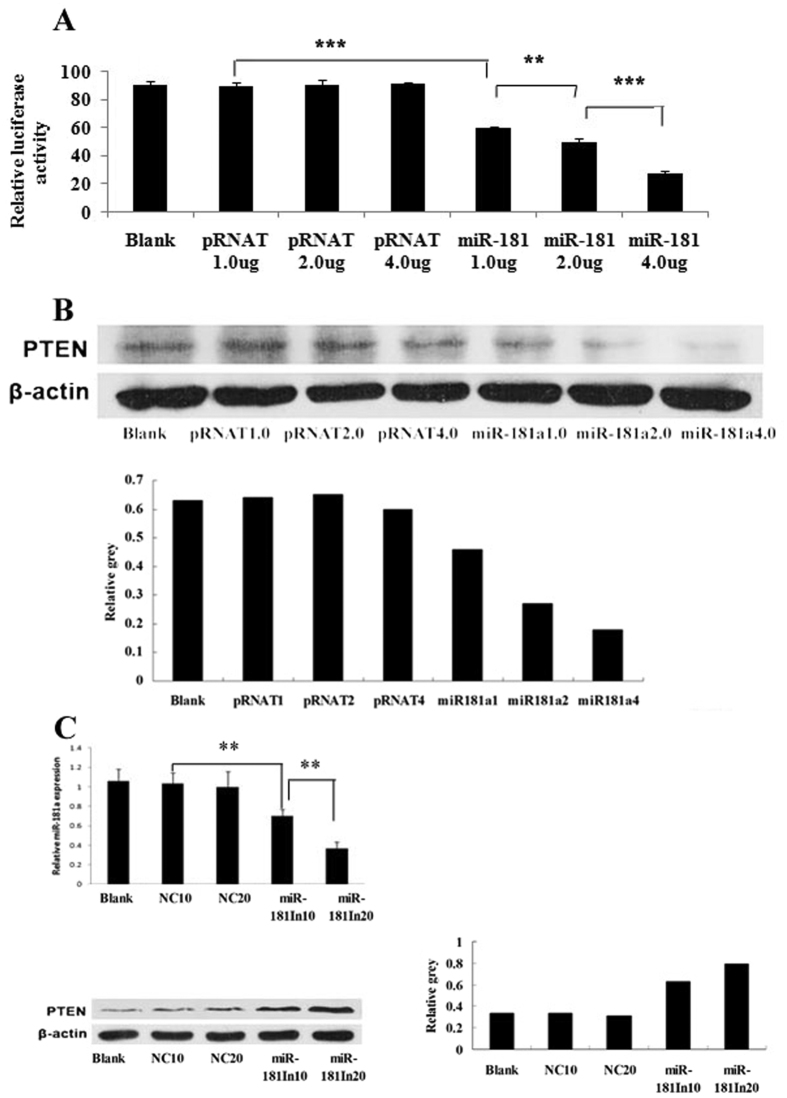
MiR-181a directly inhibits the expression of PTEN by binding 3′UTR of PTEN mRNA. (**A**) Dual-luciferase assay of PTEN-3′UTR activity in HepG2 cells transfected with miR-181a at different doses (pRNAT-miR-181a vector 1 μg, 2 μg, 4 μg, respectively) and the corresponding controls (pRNAT vector 1 μg, 2 μg, 4 μg, respectively), versus the blank group. (**B**) Western blotting analysis for PTEN in HepG2 cells transfected with miR-181a at different doses (pRNAT-miR-181a vector 1 μg, 2 μg, 4 μg, respectively) and the corresponding controls (pRNAT vector 1 μg, 2 μg, 4 μg, respectively), versus the blank group. (**C**) Western blotting analysis for PTEN in HepG2 cells transfected with miR-181a inhibitor (miR-181In10, miR-181In 20) and the corresponding controls (NC10, NC20) at different doses (10 μl, 20 μl, respectively), versus the blank group (n = 3, **p < 0.01, ***p < 0.001).

To search for the miR-181a target genes, we identified the region complementary to its seed region in the 3′UTR of human PTEN mRNA using the DIANA microT v3.0 algorithm, which was also confirmed by TargetScan and PicTar. Then, we cloned a sequence of the 3′UTR of human PTEN mRNA with the predicted target site of miR-181a into downstream of the pGL3-control luciferase reporter gene vector (pGL-PTEN). Contrasted to the negative control (pRNAT), miR-181a inhibited the luciferase activity of the pGL-PTEN in a dose-dependent manner (Fig. 3A). Correspondingly, exogenous expression of miR-181a down-regulated PTEN expression while miR-181a inhibitor promoted PTEN protein level in a dose dependent manner in HepG2 cells (Fig. 3B,C).

### MiR-181a inhibitor abolished the inhibitory effect of HBx on PTEN 3′UTR and PTEN protein expression

**Figure 4:**
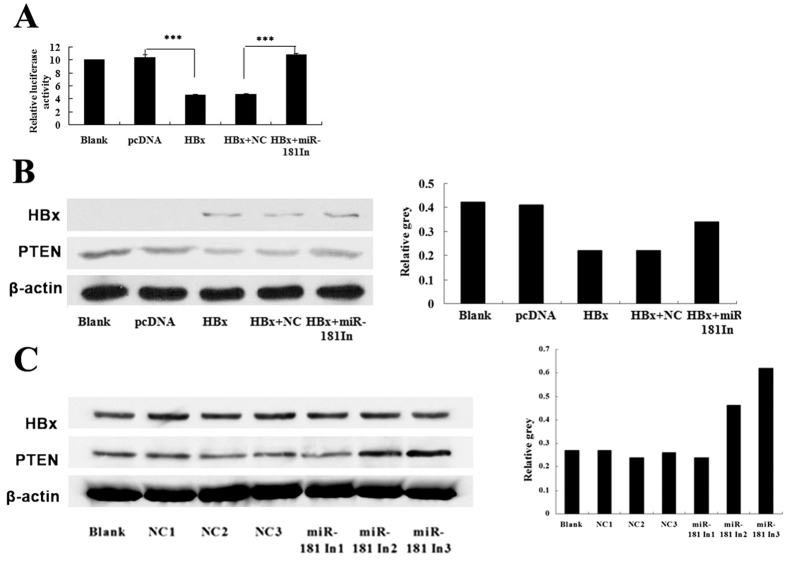
MiR-181a inhibitor abolished the inhibitory effect of HBx on PTEN 3′UTR and PTEN protein expression. (**A**) Dual-luciferase assay of PTEN-3′UTR activity in HepG2 cells transfected with pHBx and the corresponding control (pcDNA3.0), cotransfected with pHBx and miR-181a inhibitor (HBx + miR-181In) and the corresponding control (HBx + NC), versus the blank group (*p* < 0.001). (**B**) Western blotting analysis for PTEN in HepG2 cells transfected with pHBx and the corresponding control (pcDNA3.0), pHBx cotransfected with miR-181a inhibitor (HBx + miR-181In), pHBx cotransfected with the corresponding control of miR-181a inhibitor (HBx + NC), versus the blank group. (**C**) Western blotting analysis for PTEN in HepG2.2.15 cells and the cells transfected with miR-181a inhibitor (miR-181In1, miR-181In2, miR-181In3) and the corresponding controls (NC1, NC2, NC3) at different doses (0.125 pM, 0.25 pM, 0.5 pM, respectively), versus the blank group (n = 3, ***p < 0.001).

Dual-luciferase assay revealed that exogenous expression of HBx led to by up to 44% reduction of pGL-PTEN luciferase activity compared with the control group in HepG2 cells (*p* < 0.001). However, miR-181a inhibitor reversed the decreased luciferase activity of pGL-PTEN induced by HBx (Fig. 4A). Furthermore, to verify the role of miR-181a in the regulation of PTEN by HBx, we analyzed the PTEN protein level in HepG2 cells co-transfected with pHBx and the specific miR-181a inhibitor (miR181a-IN). Contrasted to the negative control (miR181a-NC), miR-181a inhibitor rescued the suppressed PTEN expression resulted by HBx (Fig. 4B). Similarly, miR-181a inhibitor promoted PTEN expression in a dose-dependent manner in HepG2.2.15 cells (Fig. 4C).

### HBx inhibits cell apoptosis through up-regulates miR-181a and in turn down-regulates PTEN

**Figure 5:**
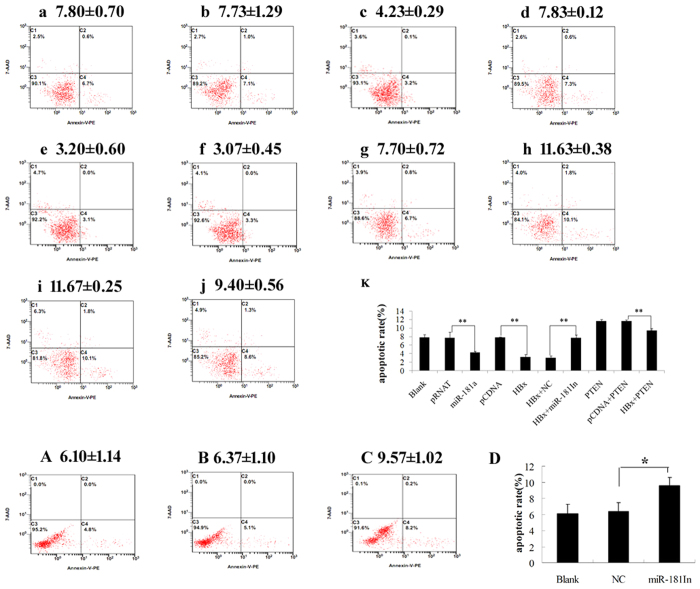
HBx inhibits cell apoptosis through up-regulates miR-181a and in turn down-regulates PTEN. Cell apoptosis was analyzed by using the Annexin V-PE apoptosis detection kit. Apoptosis rate of HepG2 cells transfected with miR-181a and the corresponding controls, versus the blank group (**a** Blank, **b** pRNAT, **c** miR-181a). Apoptosis rate of HepG2 cells transfected with pHBx and the corresponding control (**d** pcDNA3.0, **e** HBx), cotransfected with pHBx and miR-181a inhibitor and the corresponding control (**f** HBx + NC, **g** HBx + miR-181In). Apoptosis rate of HepG2 cells transfected with PTEN, pHBx cotransfected with PTEN, pcDNA3.0 cotransfected with PTEN (**h** PTEN, **i** pcDNA + PTEN, **j**. HBx + PTEN). **k** Histogram of the apoptosis rate from group **a** to **j**. Apoptosis rate of HepG2.2.15 cells transfected with miR-181a inhibitor (miR-181In) and the corresponding controls (NC), versus the blank group (**A** Blank, **B** NC, **C** miR-181In). (**D**) Histogram of the apoptosis rate from group **A** to **C**. (n = 3, * *p* < 0.05, **p < 0.01).

We then investigated the biological significance of the elevated miR-181a in hepatocyte lines. Cell apoptosis is a key character during tumorigenesis. The miR-181a or its negative control vector (pRNAT) was transiently transfected into HepG2 cells. Expression of miR-181a was verified by qRT-PCR which revealed the transfection of miR-181a restoring its expression in HepG2 cells (data had not been shown). Analysis of apoptosis was performed after culturing for 48 h by using the Annexin V-PE apoptosis detection kit. The results showed that the apoptosis rate of miR-181a-transfected cells was lower than that of negative control and the blank group (4.23% ± 0.29% *vs* 7.73% ± 1.29% and 7.80% ± 0.70%, respectively, *p* < 0.001). Similar effects were found in pHBx-transfected HepG2 cells (3.20% ± 0.60%) compared with the corresponding control (7.83% ± 0.12%) (*p* < 0.001). However, miR-181a inhibitor reversed the decreased apoptosis rate of pHBx-transfected cells (increased from 3.07% ± 0.45% to 7.70% ± 0.72%, *p* < 0.001). Meanwhile, PTEN promoted the apoptosis rate of HepG2 cells while HBx inhibited the apoptotic effect of PTEN (*p* < 0.01). As for HBV expressing cells, miR-181a inhibitor also promoted the apoptosis rate of HepG2.2.15 cells (Fig. 5).

### HBx promotes cell proliferation activity via up-regulating miR-181a and down-regulating PTEN

**Figure 6:**
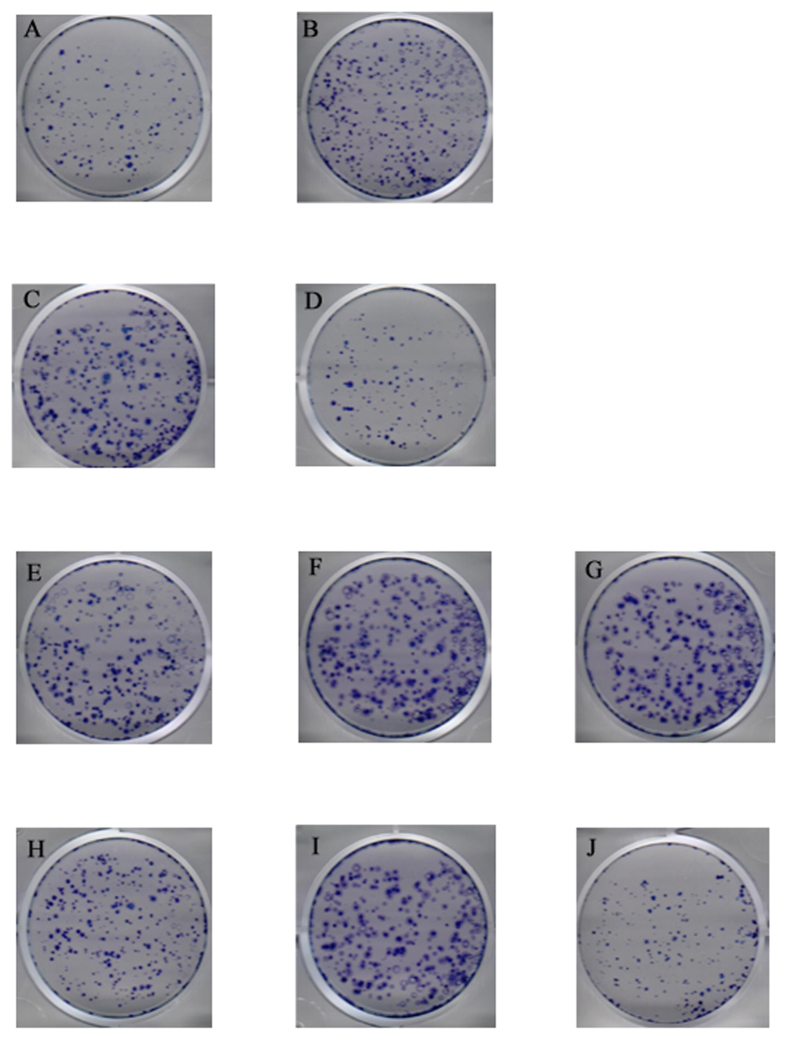
HBx promotes cell proliferation activity via up-regulating miR-181a and down-regulating PTEN. The clone formation test of HepG2 cells transfected with miR-181a and the corresponding controls, cotransfected with miR-181a and the mutation vector of PTEN, cotransfected with miR-181a and PTEN (**A** pRNAT, **B** miR-181a, **C** miR-181a + PTEN-mut, **D** miR-181a + PTEN). The clone formation test of HepG2 cells transfected with pHBx and the corresponding controls, cotransfected with pHBx and miR-181a inhibitor (miR-181In) and the corresponding controls, cotransfected with pHBx and the mutation vector of PTEN, cotransfected with pHBx and PTEN (**E** pCDNA3.0, **F** HBx, **G** HBx + NC, **H** HBx + miR-181In, **I** HBx + PTEN-mut, **J** HBx + PTEN).

**Figure 7:**
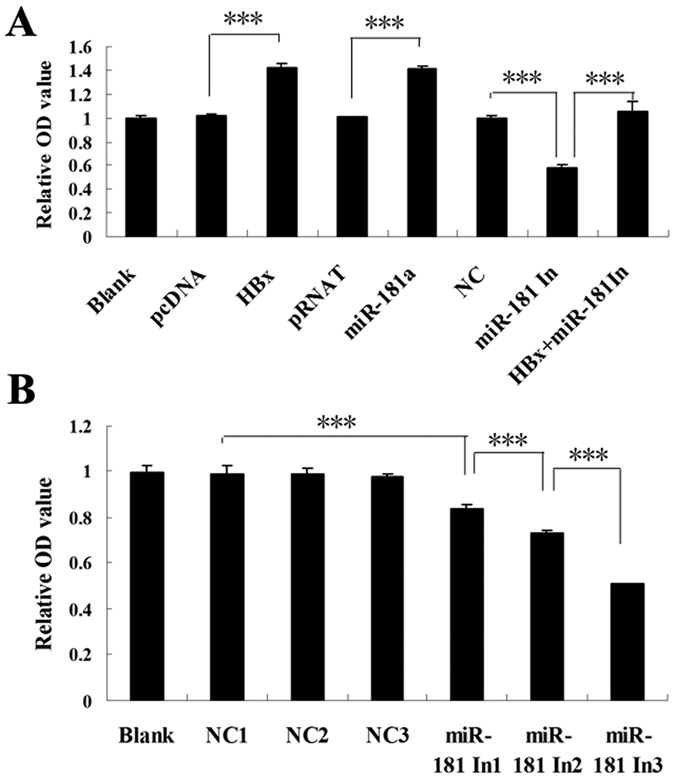
Cell viability assay (MTT assay) reveals the effect of HBx and miR-181a on cell proliferation. (**A**) MTT assay for HepG2 cells transfected by pHBx, miR-181a, miR-181a inhibitor (miR-181In) and corresponding controls, and cells cotransfected with pHBx and miR-181a inhibitor (miR-181In), versus the blank group. (**B**) MTT assay for HepG2.2.15 cells transfected with miR-181a inhibitor (miR-181a In1, miR-181a In2, miR-181a In3) and the corresponding controls (NC1, NC2, NC3) at different doses (0.125 pM, 0.25 pM, 0.5 pM, respectively), versus the blank group (n = 3, ***p < 0.001).

The proliferation activity of miR-181a on cells was detected by clone formation test and MTT assay. The result showed that the clone formation rate of miR-181a-transfected cells was significantly higher than that of negative control (10.08% *vs* 1.61%). Contrasted to the mutation vector of PTEN (pPTEN-mut), PTEN reversed the proliferation activity of miR-181a on cells more significantly (10.45% *vs* 1.60%) (Fig. 6A,B,C,D). The clone formation rate of pHBx -transfected cells was higher than that of control group (16.72% *vs* 10.37%) (Fig. 6E,F). However, miR-181a inhibitor suppressed the clone formation rate of the former (7.52% *vs* 17.80%) (Fig. 6G,H). These results demonstrate that miR-181a play a vital role in promoting cell proliferation activity induced by HBx. Moreover, compared with pPTEN-mut, PTEN eliminated the effect of HBx on cells proliferation more remarkably (16.68% vs 1.55%) (Fig. 6I,J). MTT assay revealed that the proliferation activity of cells transfected by pHBx or miR-181a was significantly higher than that of corresponding control (*p* < 0.001). MiR-181a inhibitor abolished the proliferating effect of HBx on HepG2 cells. Besides, miR-181a inhibitor suppressed the proliferation activity of HepG2.2.15 cells in a dose-dependent manner (Fig. 7A,B).

## Discussion

HBV is widely accepted to be a main cause of HCC. HBx, which is recruited on cellular chromatin and modulates chromatin dynamics at specific gene loci, plays an important role in the progression of HBV-associated HCC^13^,^14^,^15^. HBx has been shown to induce various signaling pathways and cellular proteins that could link HCC with HBV infection. In recent years, miRNAs have been reported frequently to be involved in tumorigenesis^16^. MiR-181a was up regulated in EpCAM + AFP + hepatic cancer stem cells (HepCSCs)^8^,^9^. PTEN was down-regulated in HBV infected cells and HCC. PTEN under-expression is associated with larger tumor size, tumor microsatellite formation, and shorter overall survival in patients with HCC^11^. We revealed that miR-181a was the most elevated miRNA in HepG2.2.15 cells compared with HepG2 cells, and PTEN was down-regulated in HBV expressing cells. Then, we identified HBx played a major role in up-regulation of miR181a and suppression of PTEN in HBV expressing cells. Furthermore, we explored the role of miR-181a in the regulation of HBx on PTEN. We found that miR-181a involved in the effect of HBx on PTEN 3′UTR activity and PTEN protein expression. All this indicate that HBx induces the down-regulation of PTEN, at least in part, via inducing miRNA-181a during HBV infection.

To identify the possible mechanism of HBx up-regulating miR-181a, we designed the promoter of miR-181a according to the literature^10^ and reconstructed the miR-181a promoter construct pGL3-miR-181a-P. The results revealed that HBx induced miR-181a up-expression by enhancing its promoter activity. The miR-181a promoter designed in this research contains cyclic adenosine monophosphate response element-binding protein element (CREB), a transcription factor binding site which could be bound by HBx^17^,^18^. It is reasonable to propose that HBx enhance the activity of miR-181a promoter via binding to the CREB site. However, further studies should be taken to identify its probability.

In colon cell line, up-regulation of miR-181a suppressed PTEN expression, activated phosphorylation of AKT (p-AKT), promoted cell viability, and inhibited apoptosis^19^. We identified PTEN as a direct target gene of miR-181a in hepatocyte line. This is the very first report demonstrating the regulatory mechanism of miR-181a and PTEN in hepatic cells and HBV expressing cells. We also found that HBx disturbs the cross- talk between miR-181a and PTEN. MiR-181a is a key modulatory factor for cell proliferation and differentiation at both transcriptional and posttranscriptional levels in gastric cancer, acute myeloid leukemia^20^,^21^. MiR-181a overexpression resulted in promotion of cell proliferation and migration but inhibition of apoptosis in colorectal cancer^22^. HBV plays an important role in promoting cell growth^23^,^24^. We revealed that HBx promoted hepatoma cell proliferation, inhibited cell apoptosis by up-regulating miR-181a and consequently suppressing PTEN, which revealed a novel mechanism for the growth- promoting effect of HBV on hepatoma cell. Next, we will explore the effect and mechanism of miR-181a on the activation of PI3K and Akt in hepatocellular cell lines to further support the pro-proliferation and anti-apoptotic activity of miR-181a.

MiR-181 family members were up-regulated in HepCSCs. Moreover, miR-181 family members were highly expressed in embryonic livers and in isolated hepatic stem cells^8^. However, the mechanism of miR-181 regulating EpCAM expression is not quite clear. EpCAM was a Wnt-beta-catenin signaling target gene and activation of the Wnt-beta-catenin pathway promoted EpCAM expression in cultured normal human hepatocytes and HCC cell lines^25^. Moreover, the loss of PTEN induced the activation of the beta-catenin pathway^26^. Our research confirmed the inhibiting effect of miR-181a on PTEN expression. We propose that the promotion of miR-181 driven by HBx in HCCs could influence the beta-catenin pathway and promote EpCAM expression by suppressing PTEN protein expression, which may have revealed a novel regulatory link between miR-181a and human EpCAM(+) liver cancer stem/progenitor cells.

Collectively, our results have revealed a new mechanism of HBV-related HCC. We delineate the role of HBx on the expression of miR-181a and its role in inducing the proliferation and antiapoptosis of hepatoma cells. HBx induces miR-181a expression by enhancing its promoter activity, and disturbs the cross- talk between miR-181a and PTEN. Moreover, miR-181a promotes cell proliferation and suppresses apoptosis by targeting PTEN. These findings indicate that miR-181a plays a vital role in the regulation of HCC cell proliferation and functions as an onco-miRNA in HBV-related HCC. Targeting miR-181a may provide an effective therapeutic approach to eradicate HBV-related HCC.

## Materials and Methods

### Construction of vectors

The luciferase expression plasmid (pGL3) driven by miR-181a promoter was constructed by cloning miR-181a promoter cDNA into the pGL3 vector, named pGL-181a-P. The miR-181a promoter cDNA was generated from HepG2 cell genomic DNA. The primers used to amplify the sequence (−800~+240) were 5′-ACGGTACCTGCAGGATCTCAGCAAAGGA-3′ (forward) and 5′-ACCTCGAGAGGAA CAGTGAGCAGTAGGA-3′ (reverse)^10^. pRNAT-miR-181a vector, which can stably express of miR-181a, contains pri-miR-181a and some of its flanking sequences, and the sequences were amplified by the following primers: 5′-CGATGGATCCTGAGTTTTGAGGTTG-3′ (forward) and 5′-ATCGAAGCTTAAA AAATGGA GTAGATGATGG-3′ (reverse).The PCR product was subcloned into a pRNAT-U6.1/Neo vector (Promega, Madison, WI, USA), referred to as miR-181a vector. The 3′untranslated regions (3′-UTRs) of PTEN containing an intact miR-181a recognition sequence were amplified by PCR from human genomic DNA, and the PCR product was subcloned into a pGL3-control vector (Promega) immediately downstream of the luciferase gene. The primers used were 5′-CGTTCTAGAACTTCATGTTTTGAAGAT-3′ (forward) and 5′-GGGGGCCGGCC AGTCATAGCAATTCTTGT-3′ (reverse). This plasmid was named pGL-PTEN. MiR-181a inhibitor (miR181a-In) and its negative control (NC) were purchased from Gene Copoeia (Rockville, Md, USA).The PTEN-expressing plasmid was cloned by PCR from human cDNA (the reverse transcription product from human hepatic cell line QSG7701) using the following primers: 5′-TCGAAGCTTATGACAGC CATCATC-3′ (sense) and 5′- CCGTCTAGATCAGA CTTTTGTAAT-3′ (antisense). pcDNA3.1 (pcDNA) was used as the expression vector. The expression plasmid was named pPTEN. The mutation vector of PTEN (pPTEN-mut) was kindly presented by Doctor Du Cheng^27^. The plasmids expressing the four proteins of HBV (HBx protein, surface antigen (HBsAg), core protein (HBcAg), and DNA polymerase protein (HBp)) were cloned using PCR from pHBV1.3, which was kindly presented by Doctor Songdong Meng from the Chinese Academy of Sciences (CAS)^28^. The pcDNA3.1 (pcDNA) was used as the expression vector. The four plasmids were named pHBx, pHBs, pHBc, and pHBp, respectively. SiRNA targeting HBx mRNAs was designed according to the GenScript siRNA Target Finder (https://www.genscript.com/sslbin/app/rnai). The sense and antisense oligonucleotides, which constituted the template for generating the siRNAs, were subcloned into the pRNAT-U6.1/Neo vector with the U6-RNA promoter between the HindIII and BamHI restriction sites. The last sequences selected for gene silencing in our study were 5′-TTCACCTCTGCACGTTGCA-3′ (sense) and 5′-TGCAACGTGCAGAGGTGAA-3′ (antisense). The HBx siRNA-expressing plasmid was named pSiHBx.

### Cell Culture and Transfection

The HCC cell lines, HepG2 (HBV negative) and HepG2.2.15 (HBV positive) were maintained in Dulbecco’s modified Eagle’s medium (DMEM, Hyclone, Shanghai, China) supplemented with 10% fetal bovine serum (FBS, Gibco, New York, USA) containing 100 U/ml penicillin and streptomycin at 37 °C in a humidified atmosphere with 5% CO2.

For transfection with plasmid DNA, cells were plated in 6-well plates in antibiotic free growth medium at a density of 1 × 10^6^ cells/well. After 24 hours, the transfection was performed using Lipofectamine 2000 according to the manufacturer’s instructions. The growth medium was changed after 6 h. Transfected cells were harvested at 48 hours and the total cellular RNA or protein was isolated for the next experiment.

### MicroRNA microarray and qRT-PCR

Total RNA was isolated using TRIzol reagent (Invitrogen, Carlsbad, CA, USA). The microRNA gene microarray (Affymetrix microRNA 2.0 array) was completed by Capital Bio Corporation. Complementary DNA was synthesised from total RNA (both mRNA and microRNA) using the TaKaRa One step PrimeScript® miRNA cDNA Synthesis Kit (Perfect Real Time) according to the manufacturer’s instructions. To quantify the target mRNA or miRNAs, quantitative real-time polymerase chain reaction (qRT-PCR) was performed using the ABI 7500 Real-Time PCR System with Takara SYBR_Premix Ex TaqTM II (Perfect Real Time) according to the manufacturer’s instructions. The forward primers of each target mRNA or miRNA are described as follows: β-actin:5′-CCAACTGGGACGACAT-3′ (sense) and 5′-AGCCTGGATAGCAACG-3′ (antisense); PTEN: 5′-GGACGAACTGGTGTAA-3′(sense) and 5′-CCTCTGACTGGGAATAG-3′ (antisense); HBx: 5′-TCTGTGCCTTCTCATCTGC-3′ (sense) and 5′TCGGTCGTTGACATTGCTG-3′ (antisense); miR-181a: 5′-GCCGAAACATTCAACGCTGTC-3′ (sense) and 5′-GTGCAGGGTCCGAGGT-3′ (antisense); RT primer of miR181a: 5′-GTCGTATCCAGTGCA GGGTCCGAGGTATTCGCACTGGATACGACACTCACC-3′. U6 was used as a miRNA internal control, the primers for U6 were: 5′-CGCTTCGGCAGCACATATAC-3′ (sense) and 5′-TTCACGAATTTGCGTGTCAT-3′ (antisense). The relative expression of miRNA or mRNA was analyzed using the ΔΔCt method. ΔCt was calculated by subtracting the Ct of U6 (for miRNA) or β-actin (for mRNA) RNA from the Ct of the miRNA or mRNA of interest. The ΔΔCt was calculated by subtracting the ΔCt of the reference sample from the ΔCt of each sample. The fold change was determined using the equation 2−ΔΔCt.

### Dual-luciferase assay

To study the effects of HBx and miR-181a on the activity of 3′untranslated regions (3′-UTRs) of PTEN which contains an intact miR-181a recognition sequence, the expression plasmids for HBx or pcDNA, 3′untranslated regions of PTEN-luciferase reporter plasmids (pGL-PTEN) and Renilla luciferase (RL, Promega) expression vector (PRL-TK) were mixed (4:0.5:0.1) and co-transfected into HepG2 cells cultured in 6-well plates; the incremental dose of miR-181a or pRNAT vector (1,2,4 μg, respectively) was co-transfected with pGL-PTEN (0.5 μg) and PRL-TK(0.1 μg) into HepG2 cells. To investigate the effects of HBx on the activity of the promoter of miR-181a, the expression plasmids for HBx or pcDNA, pGL181a-P and PRL-TK were mixed (4:0.5:0.1) and co-transfected into HepG2 cells cultured in 6-well plates. The cells were split, and the FL/RL activities were measured 48 h after transfection using the Dual-Luciferase Assay Kit (Promega).

### Western blotting analyses

Cells were harvested and lysed in 500 μl cell lysis reagent according to the protocol provided by the manufacturer. Cell lysates were then clarified by centrifugation, and the protein concentration of each sample was determined using the BCA Protein Assay Kit. Standard western blotting procedures were performed. Membranes were blocked in PBS-0.1% Triton x-100 (PBS-T)/3% (w/v) milk for 12 hr at 4 °C. Membranes were then incubated with primary antibodies diluted in PBS-T/3% (w/v) milk for 2 hr at 37 °C. Subsequently, membranes were washed with PBS-T three times (5 min × 3) and incubated with peroxidase-conjugated secondary antibody diluted in PBS-T/3% (w/v) milk at 37 °C for 2 hr. Membranes were washed in PBS-T and antibody/antigen complexes were detected using the Super Signal West Pico Chemiluminescent Substrate (Thermo, Rockford, USA). The primary antibodies were mouse monoclonal anti-PTEN (Santa Cruz, USA), mouse monoclonal anti-HBx (Abcam, Cambridge, UK) and mouse monoclonal anti-b-actin (Sigma, St. Louis MO, USA). The secondary antibodies were goat anti-mouse IgG-HRP (Thermo). Expression levels of PTEN was semi-quantitatively analyzed by using Image J software, normalized to β-actin density; versus the blank group.

### Flow cytometry analysis

Flow cytometry was used to quantitate the percentage of apoptotic cells. For transfection with plasmid DNA, cells were plated in 6-well plates in antibiotic free growth medium at a density of 1 × 10^6^ cells/well. After 24 hours, the transfection was performed using Lipofectamine 2000 according to the manufacturer’s instructions. The growth medium was changed after 6 h. Transfected cells were harvested at 48 hours. Apoptosis incidence was detected by using the Annexin V-PE apoptosis detection kit.

### Colony-forming unit assay

Clone formation test was conducted to confirm malignant transformation. HepG2 cells were seeded into 6-well plates as 2000 cell/well with triplicate and incubated at 37 °C, 5% CO2 for 24 h. Then the plasmids were transfected into HepG2 cells. The growth medium was changed after 6 h from transfection. The cells were continued to be incubated for 14 days and the visible cloning were observed in the dish. Culture medium was discarded. Each well was washed with PBS twice carefully. Then cells were fixed with 4% paraformaldehyde for 10 min and stained with trypan blue. After washed with running water three times and dried at room temperature, each well was observed and photographed. Cell colonies with more than 50 cells were counted under the microscope. Clone formation rate was calculated as following formula: Clone formation rate = number of formed colony/number of seeded cells ×100%.

### Cell viability assay (MTT assay)

HepG2 or HepG2.2.15 cells were seeded into 96-well plates as 2000 cell/well with triplicate and incubated at 37 °C, 5% CO2 for 24 h. Then the plasmids were transfected into cells. The growth medium was changed after 6 h from transfection. The cells were continued to be incubated for 48 h and were added 20 μl MTT regent (5 mg/ml) per well. The cells were incubated for 4 h until purple precipitate was visible. Culture medium was discarded. Each well was added 150 μl DMSO and was followed by 10 min oscillation. The absorbance in each well, including the blanks was measured at 570 nm in a multimode plate reader (EnSight^TM^). The relative OD values of experimental group versus the blank group were recorded.

### Statistical analysis

All statistical analyses were carried out using the SPSS16.0 statistical software package. Continuous variables were expressed as mean ± SD. Differences between groups were calculated with Student’s t test. A two-tailed *P* value test was used with a *P* value of <0.05 considered statistically significant.

## Additional Information

**How to cite this article**: Tian, Y. *et al*. HBx promotes cell proliferation by disturbing the cross-talk between miR-181a and PTEN. *Sci. Rep.* **7**, 40089; doi: 10.1038/srep40089 (2017).

**Publisher's note:** Springer Nature remains neutral with regard to jurisdictional claims in published maps and institutional affiliations.
